# Adolescents’ risky decision-making activates neural networks related to social cognition and cognitive control processes

**DOI:** 10.3389/fnhum.2014.00060

**Published:** 2014-02-14

**Authors:** María José Rodrigo, Iván Padrón, Manuel de Vega, Evelyn C. Ferstl

**Affiliations:** ^1^Faculty of Psychology, University of La Laguna, San Cristobal de La Laguna, Santa Cruz de TenerifeSpain; ^2^Centre for Cognitive Science, University of FreiburgFreiburg, Germany

**Keywords:** adolescent risk and ambiguous decision-making, dangerous and safe choices, fMRI, decision-making in social context, emotional and social cognitive processing, age and gender differences

## Abstract

This study examines by means of functional magnetic resonance imaging the neural mechanisms underlying adolescents’ risk decision-making in social contexts. We hypothesize that the social context could engage brain regions associated with social cognition processes and developmental changes are also expected. Sixty participants (adolescents: 17–18, and young adults: 21–22 years old) read narratives describing typical situations of decision-making in the presence of peers. They were asked to make choices in risky situations (e.g., taking or refusing a drug) or ambiguous situations (e.g., eating a hamburger or a hotdog). Risky as compared to ambiguous scenarios activated bilateral temporoparietal junction (TPJ), bilateral middle temporal gyrus (MTG), right medial prefrontal cortex, and the precuneus bilaterally; i.e., brain regions related to social cognition processes, such as self-reflection and theory of mind (ToM). In addition, brain structures related to cognitive control were active [right anterior cingulate cortex (ACC), bilateral dorsolateral prefrontal cortex (DLPFC), bilateral orbitofrontal cortex], whereas no significant clusters were obtained in the reward system (ventral striatum). Choosing the dangerous option involved a further activation of control areas (ACC) and emotional and social cognition areas (temporal pole). Adolescents employed more neural resources than young adults in the right DLPFC and the right TPJ in risk situations. When choosing the dangerous option, young adults showed a further engagement in ToM related regions (bilateral MTG) and in motor control regions related to the planning of actions (pre-supplementary motor area). Finally, the right insula and the right superior temporal gyrus were more activated in women than in men, suggesting more emotional involvement and more intensive modeling of the others’ perspective in the risky conditions. These findings call for more comprehensive developmental accounts of decision-making in social contexts that incorporate the role of emotional and social cognition processes.

## INTRODUCTION

Adolescence is a developmental period characterized by decisions and actions that give rise to an increased incidence of unintentional injuries and violence, alcohol and drug abuse, unintended pregnancy and sexually transmitted diseases. Results from the Youth Risk Behavior Survey (2011) indicate that many high school students are engaged in health-risk behaviors associated with the leading causes of death among persons aged 10–24 years in the United States. Many studies have shown that risky behaviors are more frequent during adolescence and early adult years than in adults over 25 and are major contributors to physical and psychological problems (Steinberg, 2004, 2008; Eaton et al., 2008). Of special concern is the adolescents’ increasing reliance on risk-taking behavior in decision-making situations, especially in the presence of peers as compared to adults (Gardner and Steinberg, 2005). The goal of this study is to examine, by means of functional magnetic resonance imaging (fMRI), neural responses to risk and ambiguous decision-making in social scenarios involving the presence of peers in adolescents and young adults.

### RISK AND AMBIGUOUS DECISION-MAKING

In the literature of decision-making there is a common distinction between decisions under ambiguity and decisions under risk (e.g., Bechara et al., 2005). In ambiguous decisions, the probability of a specific outcome is either unknown or close to chance and the two choices do not differ in reward value nor in the likelihood of negative consequences. For example, in the two-choice prediction task, the participant chooses on which side of a house a car will appear. The probability of the car appearing on the left side of the house is identical to it appearing on the right side and there is no risk associated with choosing one side or the other. In decisions involving risk, the possible outcomes are also uncertain, but participants are asked to decide between a safe choice and a dangerous choice, given the likelihood of a given outcome. For example, in gambling tasks safe choices may have a high probability of gaining a reward, but the reward is relatively low in value. In contrast, dangerous choices may have a low probability of gaining a reward, though the reward is substantially larger in value.

Neuroimaging studies have examined whether the neural substrates of decision-making may differ depending on the nature of the decision required. A meta-analysis of fMRI studies with gambling tasks in adults (e.g., the Iowa Gambling Task, the Cambridge Risk Task) reported that ambiguous decision-making was associated with activity in the dorsolateral prefrontal cortex (DLPFC), dorsal and anterior cingulate cortex (ACC), and the parietal lobe, whereas risky decision-making was associated with activity in the orbitofrontal cortex (OFC), and the rostral portions of the ACC (Krain et al., 2006). However, some fMRI studies suggest that the role of the right DLPFC may be particularly critical for the regulation of risk-taking behavior (Ernst et al., 2002; Fishbein et al., 2005). This was confirmed by a transcranial magnetic stimulation (TMS) study in which the transient disruption of the function of the right DLPFC, but not of the left DLPFC, increased participants’ riskier decision-making in a gambling task (Knoch et al., 2006). Other studies found that ambiguity, relative to risk, increased the blood oxygen level dependent (BOLD) signal in the OFC and the amygdala, possibly since both are involved in detecting relevant stimuli of uncertain value (Elliott et al., 2000; Hsu et al., 2005; Levy et al., 2010). In sum, the specific role of the DLPFC and the OFC in risk and ambiguous decision-making is still controversial.

### RISK DECISION-MAKING IN SOCIAL CONTEXTS

Most of the studies using the fMRI technique have investigated the neural correlates of risk decision-making by using gambles or bets as decision tasks in a social vacuum (Sanfey, 2007). A few studies have investigated the neural correlates of risky decision-making when participants are confronted with a social scenario in which the presence of others may modulate their decisions. Such scenarios may require mentalizing or theory of mind (ToM) abilities to explain and predict others’ behaviors by attributing independent mental states to them, such as thoughts, beliefs, and desires (Frith and Frith, 2003). Some studies using competitive and cooperative social decision-making tasks (e.g., the Ultimate Game; Güth et al., 1982), which require inferences about the other players’ mental states suggest so. For instance, a number of fMRI studies in adults involving social tasks have shown activity in the brain’s reward system (e.g. nucleus accumbens) consistent with the desire to win monetary rewards and in the emotional system (e.g., insula and OFC) related to the unfairness of the offer. Interestingly, social decision-making tasks have also produced activations in ToM related regions [e.g., superior temporal sulcus (STS), temporoparietal junction (TPJ), and medial prefrontal cortex (mPFC)], consistent with the processing of one’s own and the other player’s actions and intentions (Montague, 2007; Burnett et al., 2011; Rilling and Sanfey, 2011). Activations in the mPFC, ACC, and TPJ have also been obtained during false-belief tasks, a classical task targeting ToM processing (e.g., Saxe and Kanwisher, 2003; Amodio and Frith, 2006; Saxe and Powell, 2006; Van Overwalle and Baetens, 2009; Young et al., 2010; Mar, 2011).

### ADOLESCENT RISK DECISION-MAKING

In recent years, a dual-system model on adolescent risk-taking derived from developmental neuroscience has suggested that the adolescents’ greater vulnerability to risky behavior is due to the temporal gap between the full maturation of two brain systems (Steinberg, 2008, 2010; Ernst et al., 2009; Somerville et al., 2010; Casey et al., 2011). The first system, which has been called the “socioemotional” incentive processing system is localized mainly in the ventral striatum (VS) and ventromedial prefrontal cortex (VMPFC) and includes also the amygdala and the OFC. This system is particularly important for valuation and prediction of potential rewards and punishments in decision-making and is operative in early adolescence. The second system, referred to as the “cognitive control” system is localized mainly in dorsolateral prefrontal cortex (DLPFC), the parietal cortex, and the ACC. This system subserves executive functions such as response inhibition (Luna et al., 2010), impulse regulation (Steinberg et al., 2008), and flexible rule use (Crone et al., 2006). It supports goal-directed decision-making and matures gradually over the course of adolescence and young adulthood. Accordingly, developmental evidence in a gambling task shows that activation in dorsal ACC showed a linear decrease with age from 8- to 10-year-old participants to adult participants associated with risky choices, probably as a reflection of the need to engage more brain activity in these areas in less mature stages of development. In turn, activation in VMPFC and VS showed an inverted U-shaped developmental pattern, with a peak in adolescence compared to children and adults (Van Leijenhorst et al., 2010).

However, recent reviews suggest that the evidence obtained in support of the dual model may not generalize to all contexts or tasks (Crone and Dahl, 2012; Pfeifer and Allen, 2012). For example, Chein et al. (2011) examined the impact of the presence of peers on risk decision-making in a driving game (Gardner and Steinberg, 2005). The presence of familiar peers heightened responses in VS and OFC during risk choices more for adolescents than adults, in line with the dual model. Brain areas associated with cognitive control (DLPFC) were less strongly recruited by adolescents than adults, and brain activity in this area did not vary with the peer manipulation. However, a different pattern of results emerged in a second study done by Peake et al. (2013) using the same task. Adolescents’ safe choices in the driving game, after an episode of social exclusion from hypothetical peers, were associated with greater activation in right DLPFC and OFC, but also with other regions implicated in ToM abilities such as the posterior cingulate cortex and precuneus (PCC/prec), mPFC, and bilateral TPJ. Therefore, some other regions of interest (ROIs) appeared to be engaged in the same decision task when involving the social exclusion of virtual peers.

### CURRENT STUDY

The present study was designed to provide further evidence on the adolescents’ neural responses to risk decision-making in social contexts following two new directions: First, decision-making was tested in the context of realistic everyday situations. We created a novel Social Context Decision task (SCDT) that consisted of short stories that describe social situations involving risk and ambiguous decision-making. The stories mentioned everyday situations in which participants accompanied by a close peer are either involved in risk situations where they have to decide between a dangerous or a safe choice (e.g., taking or refusing a drug), or in ambiguous situations where they have to select between two neutral choices (e.g., drinking coke or orange juice). Thus, we manipulated the type of decisions to be made (ambiguous or risk decision-making), with the social context held constant (peers are mentioned in the scenario in both cases). This manipulation may help to reveal the neural response specifically associated to risk decision-making in social contexts since both conditions involved decisions. Such specificity in the neural responses would be related to the different nature of the decision task. Participants in risk situations have to decide between dangerous and safe options having knowledge of what the probability of the outcome might be, whereas participants in ambiguous situations do not know the possible outcomes associated with neutral choices. Further comparison between dangerous and safe choices in risk situations can also be informative of the decision-making process in the SCDT.

The second direction taken in this study was to test developmental effects in the pattern of brain activation under risk and ambiguous conditions in our SCDT. Young men and women from two age groups (late adolescents: 17–18 years old, and young adults: 21–22 years old) participated in the fMRI study. These ages were selected for two reasons. First, the dual model postulates an imbalance between the maturation of control and reward brain circuitry during adolescence that becomes less pronounced in early adulthood years (e.g., Steinberg, 2010). Therefore, it would be relevant to examine whether there are differences in brain activation in such critical transition. Second, age comparisons can also reveal the existence of developmental changes in social decision-making in SCDT, taking into account that the emotional and social cognition processes presumably involved also showed late developments (Blakemore and Robbins, 2012; Crone and Dahl, 2012).

Based on prior empirical studies, we first predicted that our risk and ambiguous conditions in the SCDT would activate at least part of the brain circuitry that is activated for risk and ambiguous decision-making with gambling tasks (e.g., DLPFC, OFC, and ACC). More specifically, we predicted that the brain control-related regions would be more activated while making choices in the risk situations, as more engagement of executive functions may be required, than in the ambiguous situations. According to the dual-system model, risk decision-making involves response inhibition, impulse regulation and response conflict (Steinberg et al., 2008). We also predicted heightened responses in the reward-related neural regions (e.g., VS) during dangerous choices compared to safe choices, following the dual-system model (Chein et al., 2011).

Of particular interest in this study is the potential activation of other brain systems related to social cognition processes. Our SCDT presents stories in which close peers are mentioned in the decision-making scenario. We propose that decision-making in social scenarios would presumably require participants to engage both in a self-reflective process in which a decision is made regarding oneself, and a perspective-taking process in which the peers’ mental state with regard to the decision is considered. Based on this proposal we predicted the activation of regions in two overlapping brain systems: the so-called self-reflection network involving the mPFC, PCC/Precuneus and ACC (van der Meer et al., 2010) and the ToM network involving the mPFC, TPJ, and STS among other regions (e.g., Frith and Frith, 2003; Saxe and Kanwisher, 2003; Young et al., 2010). We predicted that the SCDT would activate regions in both systems more while making choices in the risk situations than in the ambiguous situations. Social decision-making in risk situations entails a complex evaluation process since participants have to model their decisions not only according to their own point of view but also taking into account how their decisions will impact the others’ point of view about them (for example, being admired, accepted or rejected by peers according to the dangerous or safe choices). By contrast, the lack of clear expectancies related to their neutral choices, especially those referring to their potential impact to the peers’ point of view, would make the self-reflection process a less complex one, as there would be less need to take into account the peers’ point of view.

A further goal of this study was to test the existence of age and gender differences in the pattern of brain activation under risk and ambiguous conditions in our SCDT. Age-related predictions are no more than tentative, given the practical absence of developmental studies using risk decision-making in social context tasks. However, we expected developmental effects in control-related regions and reward-related regions based on previous studies. Specifically we expected higher activations in the control- and reward-related regions in adolescents than in young adults, suggesting that more brain activity in functional regions is associated with less mature stages of development. As for the developmental effects in other brain regions related to social cognition, a recent meta-analysis of studies using social reasoning paradigms and self-knowledge paradigms has shown that the mPFC is often more activated in adolescents (ages 9–18 years) compared to adults, whereas the TPJ is often less activated in adolescents (ages 10–17 years) compared to adults (Crone and Dahl, 2012). Other studies have also found that adolescents exhibit enhanced reactivity in mPFC during ToM tasks, relative to adults (Blakemore, 2008, 2011; Burnett et al., 2008; Pfeifer et al., 2009; van den Bos et al., 2011; Blakemore and Robbins, 2012; Moor et al., 2012; Pfeifer and Blakemore, 2012). However, there is an open debate about the direction of the age effects and their interpretation, since some studies have found more activation in adolescence than in young adulthood and others just the reverse pattern (Crone and Dahl, 2012; Pfeifer and Allen, 2012). Therefore, the present results may provide further evidence on that issue.

Popular gender stereotypes hold that women are more sensitive, more emotional, better mind readers, and – most importantly – less prone to risky behavior than men. However, laboratory decision-making tasks do not consistently confirm the latter claim. Men and women behave similarly in gambling risk-taking tasks (Gardner and Steinberg, 2005; Galvan et al., 2007; Van Leijenhorst et al., 2010). None of the neurological studies in social decision-making have investigated gender effects. Therefore, in this study we explored whether there are differences in neural activation between gender groups in the SCDT.

## MATERIALS AND METHODS

### PARTICIPANTS

Sixty healthy participants with no history of psychiatric illness were included in the study. We recruited participants from two age groups: thirty late adolescents (aged 17–18 years; 15 female and 15 male; mean age 17.50, SD = 0.51) from public high schools and 30 young adults (aged 21–22; 15 female and 15 male; mean age 21.40, SD = 0.49) from university and technical schools. All participants had normal or corrected-to-normal vision, and were right-handed according to the Edinburgh Handedness Inventory (Oldfield, 1971). Data for 31 additional participants were excluded for the following reasons: fourteen for excessive head movement (>1 mm), one for asymptomatic brain abnormality found in MR scan, six for incomplete experimental session, five for technical problems, and five for getting outlier scores in the screening tests (see below). All participants, or a primary caregiver in the case of minors, gave informed consent. All the procedures were approved by the Committee for Research Ethics and Animal Welfare at the University of La Laguna.

All participants were within the normal range of cognitive and verbal abilities within the cut-off values of ±2 SD in the following screening tests: (a) the Wisconsin Card Sorting Test of executive functioning: *M* = 100.9; SD = 6.4 (Berg, 1948; Grant and Berg, 1948); (b) the Working Memory-Sentences Test: *M* = 2.2; SD = 0.7 (Siegel and Ryan, 1989); and (c) the Controlled Oral Word Association Test (Phonemic Fluency: *M* = 37.1; SD = 8.8, Semantic Fluency: *M* = 24.6; SD**= 4.9, and General Fluency *M *= 30.8; SD = 5.9, Benton et al., 1994). There were no differences due to age and gender in any of the tests.

### THE SOCIAL CONTEXT DECISION TASK

The study used a novel SCDT involving two types of verbal materials: 40 risk situations and 40 ambiguous situations. We performed two normative studies for the elaboration of the verbal materials, using different participants than in the main fMRI study. The first study was designed to select the most common risk situations in the adolescents’ and young adults’ personal experience. Sixty-three risk scenarios were written, based on situations selected from the Youth Risk Behavior Survey (2011). They belonged to five domains: behaviors that contribute to unintentional injuries (i.e., jumping to the sea from a high rock), risky sport practice (i.e., climbing without appropriate equipment), unhealthy behaviors (i.e., competing to demonstrate who can eat more burgers), unprotected sexual behaviors (i.e., having sex without condom), and alcohol and other drug use (i.e., consuming cocaine). Sixty participants (half adolescents and half young adults of both gender) had to judge if they had been involved in a similar situation, they had eye witnessed it or none of them. Then, they were given examples of dangerous and safe options for each situation, and asked to rate on a 1–5 point scale how dangerous these actions would be for the protagonist.

Based on the results of the ratings, 40 risk situations (40% self-experienced and 60% eye witnessed) distributed among the four domains. The domain of sexual behaviors was excluded, as it produced the largest differences between responses given by women and men. We selected those choices scoring at the extremes of the scale (Mean = 4.03; SD = 0.48 for the dangerous option; Mean = 1.57, SD = 0.37 for the safe option). Sixty ambiguous situations were initially created, including each two neutral options, and the participants were asked to choose between them. Only those situations in which the two options were selected equal number of times (about 50%) by the two age groups and genders were included (40 ambiguous situations). The pairs of options were also tested for how dangerous these actions would be for the protagonist (Mean = 1.20, SD = 0.17) and eliminated those with significant age and gender differences. The length of the sentences in the scenarios was matched in the number of words (risky scenario: *M* = 20.27, SD = 3.09 and ambiguous scenario: *M* = 20.32, SD = 3.03). Unfamiliar words were avoided in all the scenarios.

The second study was designed to match the emotional valence of possible consequences for the two scenario conditions. For each scenario, a positive and a negative consequence were written. One hundred and twenty participants (half adolescents and half young adults of both genders) were presented with a list of 128 negative events (i.e., risk situations: “while smoking marijuana you feel dizzy and have to go to the doctor”; ambiguous situations: “while preparing a snack you cut your finger and bleed profusely”), and 128 positive events (i.e., risk situations: “you enjoy swimming at the beach”; ambiguous situations: “you enjoy the meal at the restaurant”). The participants rated them on a bipolar scale from -5 (very negative) to +5 (very positive). Positive events were rated as more positive in both conditions (risk situations: *M* = 3.98, SD = 0.42; ambiguous situations: *M* = 4.05, SD = 0.42) than negative events (risk situations: *M* = -4.66, SD = 0.39; ambiguous situations: *M* = -4.45, SD = 0.42). More importantly, ratings of negative events did not significantly differ between risk and ambiguous scenarios. Similarly, ratings of positive events did not significantly differ between risk and ambiguous scenarios.

**FIGURE 1:**
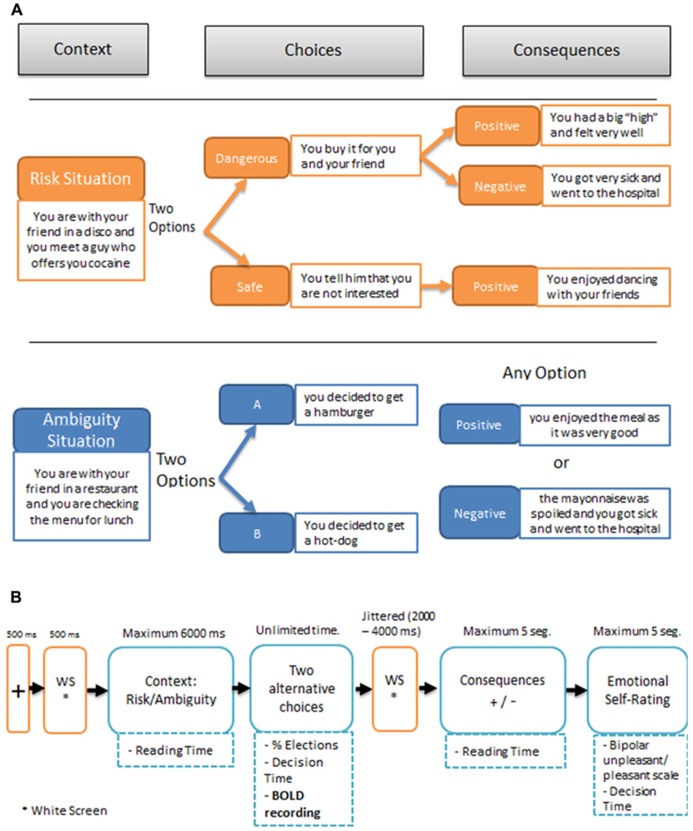
**(A)** Examples of SCDT (Social Context Decision Task), in risk and ambiguous scenarios. **(B)** Temporal sequence of events in a trial.

Both the risk and the ambiguous experimental trials involved the same sequence of events illustrated in **Figure 1A**: (1) A second-person scenario describing “you” as accompanied by a close friend; (2) The presentation of the two alternative options for the decision-making task in that scenario; (3) After the participants had made their choice, the presentation of the consequence on the screen. This consequence was either positive or negative following a pre-established table of contingencies that participants were unaware of; (4) Participants were then asked to indicate “how do you feel about what just happened?” using a linear rating scale at the bottom of the screen: from -5 (extremely bad) to +5 (extremely good). No information had to be learned or retrieved over consecutive trials.

**Figure 1B** illustrates the timing of the events in a SCDT trial, including the fMRI recording event. The presentation of each piece of information was self-paced, allowing for the registering of the reading times of the initial scenario, the decision time after the onset of the options, the choices, the reading time of the consequence, and the emotional rating. The first 15 experimental trials, including 10 risky and 5 ambiguous scenarios, were always followed by positive consequences, to encourage participants to choose dangerous options. The remaining 65 trials (30 risky and 35 ambiguous) were presented in pseudo-random order for each participant. Participants were not informed about the change in probability of the negative outcomes at any time during the experiment. The outcomes pre-established by the experimenter for the different conditions were as follows: in ambiguous scenarios 35% of the consequences were negative and 65% were positive; by contrast, in risky situations the choice of the dangerous option was followed by negative consequences 80% of the time and by positive consequences 20% of the time, whereas the choice of the safe option, was always followed by positive outcomes. Notice that in the ambiguous situations the amount of negative outcomes that receive the participants corresponds to the nominal probability set up by the experimenter because the two choices (A or B) are equivalent in terms of possible outcomes. This is not the case for the risk situations, since each participant can choose between the safe option (100% positive outcomes) and the dangerous option (80% negative outcomes), modifying accordingly the total amount of negative consequences. The stimulus presentation was controlled by means of a custom-developed script, developed in MatLab, using the Cogent 2000 v1.29 Software Toolbox (http://www.vislab.ucl.ac.uk/cogent.php/ provided in the public domain by the Laboratory of Neurobiology, University College London, UK).

### fMRI PROCEDURE

Before the scan, in a separate session, participants filled out the screening tests. Then, those participants that passed the screening came to the MRI laboratory and were prepared for the scanning session with the SCDT. First, the participants received extensive instructions and performed 10 practice trials in a mock scanner that simulated the environment and sounds of an actual MRI scanner. They were asked to imagine themselves (“imaging you”) as vividly as possible in each situation accompanied with a close friend and choose between the two alternative actions. They were also told that there was no need to remember the performance on previous trials, because the trials were not related, and that all trials were equally important. Finally, we informed them that “you will receive money as long as you follow our instructions” but in fact the money received at the end of the task (25€) was not contingent on the individual’s performance. Immediately after the instructions and training, participants were brought into the real scanner and the experimental session started.

### MRI DATA ACQUISITION

In total, 80 trials of the SCDT were presented (40 for each condition). Each participant completed two 18-min functional runs, with a 5-min resting period in between. Scanning was performed at the laboratories of the Magnetic Resonance Service of the University of La Laguna. Images were obtained using a standard whole-head coil on a 3.0 Tesla scanner Signa Excite HD model manufactured by General Electric (Milwaukee, WI, USA). The stimuli were presented visually via video-vision glasses compatible with MRI (Visuastim, Resonance Technology, Inc., Northridge, CA, USA). Participants used two response controls (one for each hand) compatible with Magnetic Resonance (Nordicneurolab). Participants pressed the lower right button with their right thumb to move from one screen to the next. To indicate their decision, participants pressed one of the upper buttons of the response devices with their right or left thumb to select the option that was displayed to the right or left of the screen, respectively. Also, they were instructed to use the same fingers to move the cursor left and right in the emotional rating task.

Functional data were acquired with a standard echo-planar imagining sequence (TR = 2500 ms, TE = 22.2 ms, flip angle = 90, FOV = 24 × 24, matrix 64 × 64, 3-mm-thick 40 axial slices acquired parallel to anterior–posterior commissural line, and voxel size 3.75 × 3.75 × 3) and two runs (of total 600–700 scans), for measurement of the BOLD effect. Head movement was minimized by padding. Before the functional scans, high-resolution T1-weight anatomic images were recorded (TR = 4.768 ms, TI = 650 TE = 1, 9, flip angle: 20, voxel size; 1.02 × 1.02 × 1, matrix 228 × 228, FOV 26 × 26, slice order: sequential, gap: 0).

### fMRI PRE-PROCESSING AND STATISTICAL ANALYSIS

Functional data processing and analyses were conducted using Statistical Parametric Mapping software (SPM8, developed by Wellcome Department of Cognitive Neurology, UK, implemented in Matlab 7.10, Match Works, Natick, MA). For each subject, functional images were realigned to correct head motion, corrected for slice acquisition time differences, co-registered with anatomical image, smoothed in space with a three-dimensional, 8-mm FWHM (full width at half maximum) Gaussian Kernel, and entered into a voxelwise analysis using the general linear model (GLM). A high-pass filter with a cut-off at 128 s was used to remove low frequency fluctuations. An event-related design was used and the BOLD time series data were modeled using standard hemodynamic response function (HRF) with time derivative.

The full experimental design involved two decision-making situations (risk/ambiguity) × 2 consequences (positive/negative). Decision times were used as a covariate by trial and by person because these times would influence the BOLD signal. Thus, as participants would spend more time for selecting options in the risk scenarios than in the ambiguous scenarios, the response time for each individual was included as a covariate in the analyses as a parametric modulator. This helps to control for differences in the timing between both conditions that may have some impact on the activations. The analysis focussed on the decision phase, where the risk vs. ambiguous scenarios manipulation may have more impact, by means of two contrasts: (a) the overall contrast between risk and ambiguous conditions collapsing over the choices and consequences, and (b) within the risk conditions, the contrast between dangerous and safe choices, collapsing over the consequences. We registered the BOLD signal from the onset of the two alternative choices until the participants made their decisions (see **Figure 1B**). We tested two whole-brain contrasts: Risk > Ambiguity, and Ambiguity > Risk in the whole sample of 60 participants. We also tested two whole-brain contrasts: Dangerous > Safe, and Safe > Dangerous choices in the whole sample of 60 participants. As participants would spend more time for selecting dangerous options than safe options, the response time for each individual was included as a covariate in the analyses. All contrast images (beta maps) were calculated from individual-level GLM analysis, normalized to the standard Montreal Neurological Institute (MNI) brain template with a 3.75 × 3.75 × 3 voxel size, and then entered into one sample *t*-test for the group level random- effect analyses. Cluster larger than 10 voxels that reached a corrected familywise error rate (FWE) of *p* < 0.001 were considered significant. FWE correction was implemented by selecting this option in SPM8.

We analyzed time series, employing the finite impulse response (FIR) algorithm to plot the temporal course of these activations in several ROIs. The ROIs were created by overlapping the 10 mm spheres around the peak activations in the significant clusters obtained in the voxelwise analyses with the corresponding MNI anatomical regions. The overlapping areas were computed using the MarsBaR toolbox of the SPM8 (see http://marsbar.sourceforge.net/ by Brett et al., 2002).

We also explored age and gender differences, performing second-level whole-brain analyses for the interactions Age (adolescents vs. young adults) × Type of decision-making situation (risk vs. ambiguous), and Gender × Type of decision-making situation. We also explored age and gender differences, performing whole-brain analyses that combined Age (adolescents vs. young adults) × Type of Choice (dangerous vs. safe), and Gender × Type of Choice (dangerous vs. safe). Given the reduction of data for each experimental cell, and the expectable small effects of gender and age (especially in our small range of ages), we relaxed the statistical threshold in these contrasts: reported are effects with *p* < 0.001 (uncorrected) combined with an extent threshold of clustes larger than 10 voxels.

Finally, a complementary set of correlational analyses was performed to identify brain activations that reflect individual differences in risk behavior. To this end, we first obtained the percent signal change for each participant, in the significant clusters of activation, and then we correlated these activation scores with the individual percentage of risky choices in the SCDT. A similar procedure was followed with the differential peak activations between Dangerous and Safe choices in the significant clusters obtained.

## RESULTS

### BEHAVIORAL RESULTS

Reading times for the risk scenarios were significantly longer than those for ambiguous scenarios [*M *= 4883 ms, SD = 839; *M* = 4755 ms, SD = 921, respectively; *F*(1,56) = 26.7; *p *< 0.001]. There was a significant interaction of scenarios by gender [*F*(1,56) = 6.42; *p *< 0.014], due to the fact that women spent less time in reading the ambiguous contexts than men (*M *= 4520 ms, SD = 800; *M* = 4989 ms, SD = 985, respectively), but no significant gender difference was found for the reading times of the risk scenarios. Participants spent more time for selecting options in the risk scenarios than in the ambiguous scenarios [*M *= 3814 ms, SD = 730; *M* = 3423 ms, SD = 668, respectively; *F*(1,56) = 38.6; *p *< 0.001]; no significant effects of age or gender were found. Within the risk condition, participants spent significantly more time for selecting the dangerous options than the safe options (*M* = 4054 ms, SD**= 859; *M* = 3333 ms, SD = 671, respectively; *F*(1,56) = 62.29; *p *< 0.001); no significant effects of age or gender were found. Finally, participants chose the dangerous option in 34.7% of the risk situation trials (range from 19.2 to 50.2), with no age or gender differences, and they randomly chose the A and B options in the ambiguous situation trials (range from 48.4 to 51%; *t* = 0.546, *p* > 0.05), with no significant age or gender differences.

### BRAIN REGIONS INVOLVED IN RISK vs. AMBIGUOUS CONDITIONS

**FIGURE 2:**
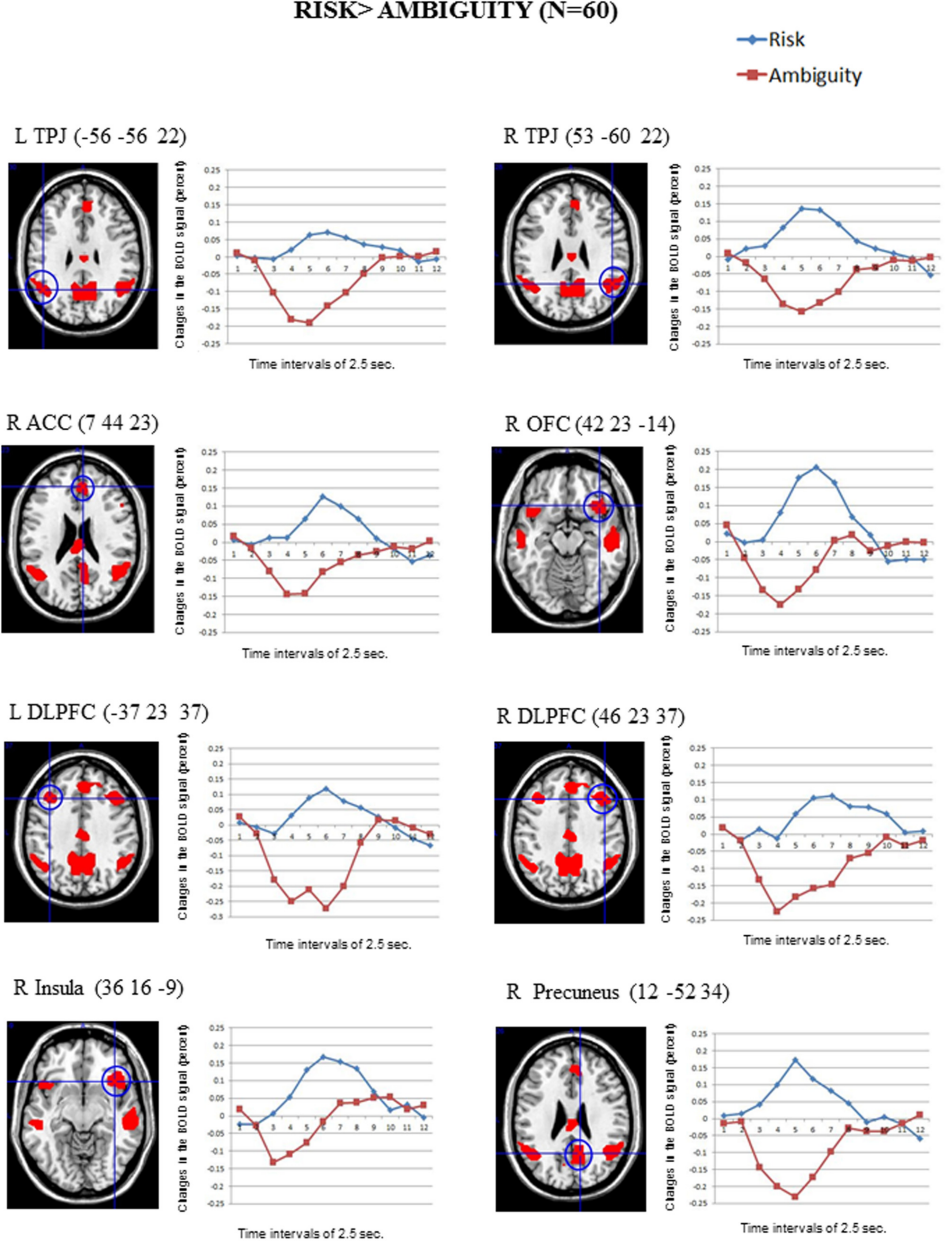
**The clusters of activation in the whole-brain analyses for the contrast *Risk* > *Ambiguity* (corrected FWE, *p* = 001 and *K* > 10; *N* = 60) are circled for several regions.** Changes in the BOLD signal (percent) across time for the same regions for the risk (blue) and the ambiguity (red) conditions are also shown, using time intervals of 2.5 s.

**Figure 2** shows the significant clusters of activation from the contrast Risk > Ambiguity in the total sample of participants. The complete list of significant clusters corrected by this covariate is shown in **Table 1**. There was a broad network of frontal and prefrontal structures activated, including right DLPFC extending to the precentral gyrus, left DLPFC, right ACC, bilateral OFC, right inferior, medial and superior frontal gyrus. In addition, there were activations in temporal and parietal structures, including the TPJ bilaterally, bilateral middle temporal gyrus (MTG), bilateral precuneus and the inferior parietal lobe. Other areas with significant clusters of activation were the right insula and the right precentral gyrus. The ROIs employed in the FIR analyses were: right ACC, right inferior OFC, left DLPFC, right DLPFC, right insula, and right precuneus. ROIs were constructed for each cluster of activation using a sphere of 10 mm around the MNI coordinates for the peak of activation. With a similar procedure we also created ROIs for the right TPJ and left TPJ, except that the MNI coordinates were taken from Saxe et al. (2009) study on ToM.

**Table 1:** Significant clusters of activation in the whole-brain analysis for the contrast Risk > Ambiguity for all participants (*N *= 60) and by age and gender groups (FWE, corrected, *p* ≤ 0.001; except contrasts marked with symbol * that employed an uncorrected threshold of *p* ≤ 0.001; extent threshold > 10 voxels).

Region	BA	Cluster size	*Z*-score	*x*, *y*, *z*
Right temporoparietal junction	37	175	7.66	53, -60, 22
Left temporoparietal junction	21	199	7.66	-56, -56, 22
Left inferior parietal lobe	40	22	6.40	-56, -51, 44
Right middle temporal gyrus	21	121	7.47	53, 1, -23
Left middle temporal gyrus	21	57	7.06	-56, -45, 1
Right inferior frontal gyrus, triangularis	45	16	5.83	53, 23, 16
Right inferior frontal gyrus, orbital	38	63	6.67	42, 23, -14
Left inferior frontal gyrus, orbital	38	14	6.10	-49, 19, -6,
Right middle frontal gyrus	44	28	5.89	46, 23, 37
Left middle frontal gyrus	44	16	5.90	-37, 23, 37
Right precentral gyrus	6	67	6.67	46, 8, 46
Right superior frontal gyrus	8	15	6.04	23, 23, 46
Right insula	48	13	6.67	36, 16, -9
Right precuneus	23	212	6.97	12, -52, 34
Left precuneus	23	63	6.75	-4, -52, 35
Right dorsomedial prefrontal cortex	9	57	6.79	5, 38, 43
Right anterior cingulate cortex	32	12	6.79	7, 44, 23
Adolescents > young adults *
Right middle frontal gyrus	9	14	4.07	38, 31, 43
Right temporoparietal junction	21	10	3.67	53, -56, 19
Female > male participants *
Right insula	48	10	3.63	38, -18, 14
Right superior temporal gyrus	42	13	3.62	57, -33, 16

*The voxel size was 3.75 mm × 3.75 mm × 3 mm.
*

The reverse comparison (Ambiguity > Risk) did not show any significant activation at the statistical threshold defined. The correlations between the activation scores in risk vs. ambiguous conditions for each participant and the percentage of the risk options in the SCDT did not reach significant values for any of the clusters in **Table 1**.

### BRAIN REGIONS INVOLVED IN DANGEROUS vs. SAFE OPTIONS IN RISK SITUATIONS

**FIGURE 3:**
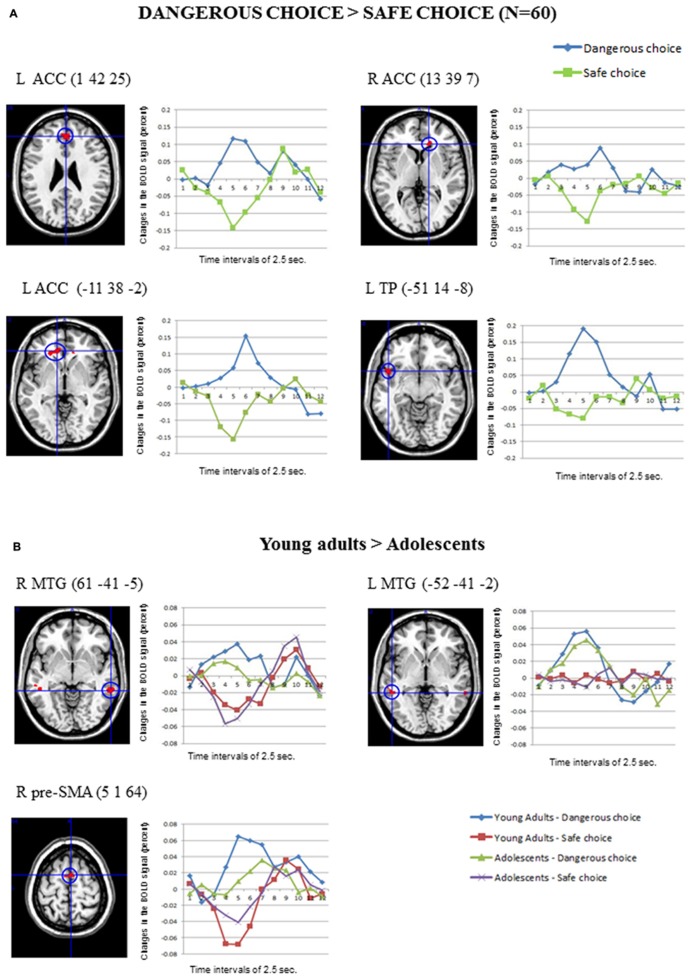
**(A)** The clusters of activation in the whole-brain analyses for the contrast *Dangerous* > *Safe choice* (uncorrected threshold, *p* = 0.0001 and *K* > 10; *N* = 60) are circled for several regions. Changes in the BOLD signal (percent) across time for the same regions for the dangerous (blue) and safe (green) options are also shown, using time intervals of 2.5 s. **(B)** Differential activations in Young adults (*N* = 30) compared to Adolescents (*N* = 30) in the contrast *Dangerous* > *Safe choice* (uncorrected threshold, *p* = 0.0001 and *K* > 10), produced two bilateral clusters in the middle temporal gyrus and the right pre-supplementary area. The changes in the BOLD signal across time for these regions in each age group are shown, using time intervals of 2.5 s.

**Figure 3A** shows the main significant clusters of activation corresponding to the contrast Dangerous > Safe choices in the total sample of participants. The complete list of significant clusters is shown in **Table 2**. There were two clusters of activation involving the left ACC, one cluster involving the right ACC and one cluster involving the left superior temporal pole (TP). The resulting ROIs employed in the FIR analyses corresponded to the same activations following the same procedure than in the previous contrast. The reverse comparison (Safe > Dangerous) did not show any significant activation at the statistical threshold defined. Finally, the correlations between the activation scores in dangerous vs. safe choices for each participant and the percentage of the risk choices in the SCDT did not reach significant values for any of the clusters in **Table 2**.

**Table 2:** Significant clusters of activation in the whole-brain analysis for the contrast Dangerous choice > Safechoice (*N* = 60) and by age groups (FWE, corrected, *p* ≤ 0.0001; except contrasts marked with symbol “*” that employed an uncorrected threshold of *p* ≤ 0.0001; extent threshold > 10 voxels).

Region	BA	Cluster size	*Z*-score	*x*, *y*, *z*
**All participants**
Left anterior cingulate cortex	32	30	4.77	1, 42, 25
Left anterior cingulate cortex	11	32	4.46	-11, 38, -2
Right anterior cingulate cortex	32	12	3.84	13, 39, 7
Left superior temporal pole	38	19	4.56	-51, 14, -8
Young adults > adolescents*
			
Right middle temporal gyrus	21	15	3.87	61, -41, -5
Left middle temporal gyrus	21	13	3.45	-52, -41, -2
Right pre-supplementary motor area	6	12	3.40	5, 1, 64

*The voxel size was 3.75 mm × 3.75 mm × 3 mm.
*

### AGE- AND GENDER-RELATED MODULATIONS OF NEURAL PROCESSES IN THE SCDT

**FIGURE 4:**
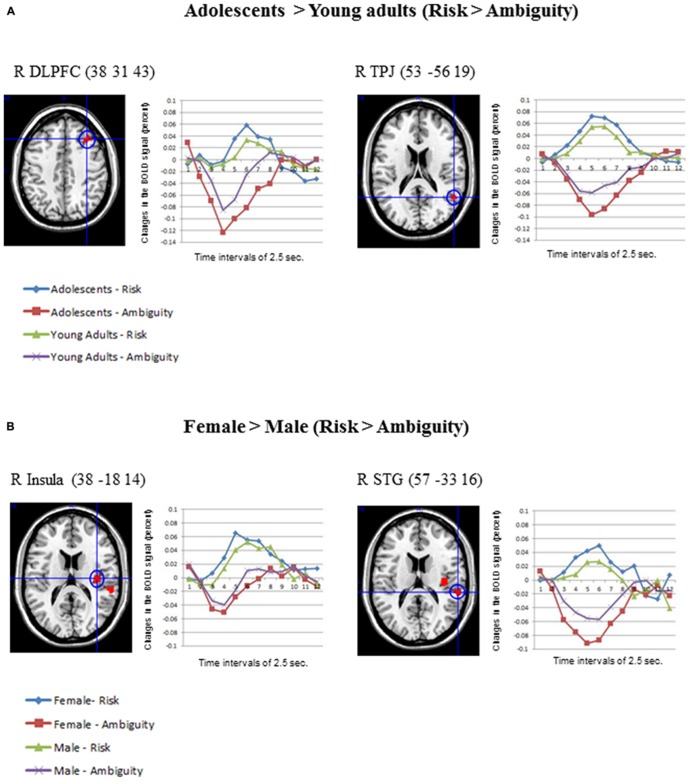
**(A)** Differential activations in Adolescents (*N* = 30) compared to Young adults (*N* = 30) in the contrast *Risk* > *Ambiguity*, produced two significant clusters in the right DLPFC and the right TPJ. The changes in the BOLD signal across time for these regions are also shown in each age group. **(B)** Differential activations in Females (*N* = 30) compared to Males (*N* = 30), in the contrast *Risk* > *Ambiguity*, produced two clusters in the right insula and the superior temporal gyrus. Also the changes in the BOLD signal across time are shown for these regions in each gender group, using time intervals of 2.5 s. Age and gender results were significant at an uncorrected threshold of *p* = 0.001, and *K* > 10.

Concerning the contrast between Risk vs. Ambiguous conditions, as **Table 1** shows, the direct contrast between the two age groups produced significant differences in the adolescents > young adults contrast for the right middle frontal gyrus (DLPFC) and the right TPJ (See **Figure 4A**). **Table 1** also shows the direct statistical contrasts testing the gender effect, which yield significant effects in the female > male comparison in the right insula and right superior temporal gyrus (see **Figure 4B**).

Concerning the contrast between Dangerous vs. Safe choices, as **Table 2** shows, the direct contrast between the two age groups produced significant differences in the young adults > adolescents contrast for the right and left MTG and the right pre-supplementary motor area (pre-SMA) in **Figure 3B**. No gender effects were found.

## DISCUSSION

This study explored the effects of type of decision in social decision-making in late adolescence and young adults. We developed a novel SCDT involving simulated situations of risk and ambiguous decision-making in which the protagonist (“you”) is accompanied by a close peer. Behaviorally, the comprehension of the scenarios and the decision-making process requires more elaboration and more cognitive cost under the risk situation than under the ambiguous situation, as suggested by the increasing reading times of the scenarios and the larger decision times. Likewise, in risk situations participants spent more time in making a dangerous choice than a safe choice. Participants selected the dangerous choice around 35% of the trials during scanning, a figure that matches well the results of the normative study in which 40% of the risk situations were self-experienced. No differences were found between the age groups, similarly to what was found in a gambling task in another study with a broader span of age groups (e.g., Van Leijenhorst et al., 2010). Women and men chose the risky options equally often, due to the avoidance of gender-biased contents in the SCDT, and consistent with other laboratory studies on decision-making (Gardner and Steinberg, 2005; Galvan et al., 2007; Van Leijenhorst et al., 2010).

Regarding the neuroimaging results for the whole sample of participants, two main findings are remarkable. First, as expected, the SCDT in risk scenarios (compared to ambiguous scenarios) activated part of the control-related circuitry usually reported in the decision-making literature employing gambling tasks, namely the right ACC, the bilateral DLPFC, the bilateral OFC as well as the right insula, associated with the emotional processes in risk scenarios. Notice that the OFC activation in risk situations cannot be due to the higher likelihood of negative consequences for the dangerous choices (80%), since participants on the average chose the dangerous option only in 34.7% of the trials, and received thus negative consequences in 27.8% of the total number of trials, in comparison with 35% of negative outcomes in the ambiguous situations. Second, as expected, and unlike the gambling tasks in non-social contexts, the SCDT in social scenarios also activated brain areas typically related to social cognition processes: the bilateral TPJ, the bilateral MTG, the right dorsomedial PFC and the bilateral precuneus. These activations are compatible with our prediction of engagement of the self-reflection network (van der Meer et al., 2010) and the ToM network (e.g., Frith and Frith, 2003; Saxe and Kanwisher, 2003). In fact, both systems greatly overlap, as the self-reflection system (specifically the dorsomedial PFC) is also responsible for evaluation and decision-making processes in self- and other-referential processing (van der Meer et al., 2010). The activation of the precuneus is also reported in both systems since this region is involved in a wide spectrum of highly self-processing operations, namely first-person perspective-taking in the experience of agency or sense of control over actions (Cavanna and Trimble, 2006) and in situation model updating during narrative comprehension (Ferstl et al., 2008). Unexpectedly, we also found activations in the right and left inferior frontal gyrus (IFG), a region primarily associated with language production (left IFG), but also with the perception of biological actions and mental inference, although its role as a potential component of the ToM network is still under discussion (Mar, 2011).

As predicted, the extensive involvement of the cognitive control-related brain regions in the SCDTs was specific to risk decision-making, appearing in the contrast Risk > Ambiguity. In contrast, the inverse comparison Ambiguity > Risk did not show any area significantly activated. This finding clearly departs from the more balanced pattern of control-related activations generally obtained for risk and ambiguity conditions in gambling tasks in non-social contexts (e.g., Krain et al., 2006). However, in line with our results, some studies also reported more involvement of control-related regions in risk-taking or conflict resolution behavior than in more neutral situations. Thus, the right DLPFC has been found to be critical for the regulation of risk-taking behavior in gambling tasks, as suggested by a study that disrupted the DLPFC function in adults by means of repetitive TMS, resulting in an increase of dangerous choices (Knoch et al., 2006). Moreover, more ACC activation has been obtained in attention and action monitoring tasks, especially in the context of response conflict, which is a similar case to our risk decision-making scenarios (Barch et al., 2000; van Veen and Carter, 2002; Wang et al., 2005; Brown and Braver, 2008; Modirrousta and Fellows, 2008). Beyond the control-related activations, we also obtained activation in the right insula in risk conditions, consistent with its role in signaling aversive consequences, which occur more frequently under risk conditions (Kringelbach and Rolls, 2004; Paulus and Stein, 2006; Clark et al., 2008).

As predicted, the SCDT activated the brain areas presumably implicated in the social cognitive processing (self-reflection and ToM networks) in the risk situations only, not in the ambiguous situations. Before reaching a conclusion it is important to acknowledge that these brain regions partly overlap with the network involved in story comprehension (Ferstl et al., 2008; Mar, 2011). Therefore, it would be the case that the pattern of findings in the SCDT would reflect the higher verbal demands required in the processing of the risk situations than in ambiguous situations. However, verbal materials in risk and ambiguous situations were controlled for their lexical and syntactical complexity, which suggests that other non-linguistic factors are involved in the activation of this neural pattern. In particular, we propose that socio-cognitive factors would be involved in the decision-making process in social contexts. Our results clearly show that merely mentioning a character is not sufficient to trigger the activation of those regions, since participants made choices in social scenarios accompanied by peers both in risk and ambiguous conditions. Notice that just being an external observer of a participant’s action is not either enough to activate them (Chein et al., 2011). Therefore, recruitment of brain regions related to social cognition processes may require thinking about a person’s beliefs that is relevant to the self-decision-making, not just acknowledging a person’s presence (Saxe et al., 2004; Uddin et al., 2007). This condition could be achieved by stressing the relevance of the peers in the decision-making after the induction of a social exclusion episode (Peake et al., 2013).

In our study, the activation of brain areas related to social cognition processes seems especially important in those risk situations requiring participants to choose between a dangerous and a safe option. We have advanced an explanation related to the nature of the type of social decision-making involved. Social decision-making in risk situations entails for the participants two sets of clear expectancies regarding: (a) the consequences for themselves of their risky or safe choices and (b) the impact of their choices to the peers’ beliefs and attitudes towards themselves. For instance, participants expect that risky choices may have negative consequences for their health but also expect to obtain positive reactions from their peers, and the reverse for the safe choices. By contrast, social decision-making in ambiguous situations did not entail such clear expectancies since the consequences of the neutral choices come by accident without any control over the situation and there are no clear expectances related to the impact of neutral choices to the peers’ point of view. Therefore, the recruitment of brain regions related to social cognition processes takes place in risk situations only, where thinking about a person’s beliefs is relevant to the self-decision-making.

An extensive involvement of the bilateral ACC region was obtained in the specific contrast Dangerous > Safe choices in the risk situations of the SCDT, consistent with previous studies on adolescent decision-making that emphasize the role of ACC in conflict processing as part of the control system (e.g., Steinberg, 2010; Van Leijenhorst et al., 2010; Chein et al., 2011). In contrast, the lack of involvement of the brain’s reward system (e.g., VS) in the SCDT is remarkable. This finding was possibly due to the lack of feedback and/or monetary reward contingent on the participants’ performance. Some researchers also observe either hypoactivity or no differences between adolescents and adults in VS responses to some reward conditions or paradigms (Pfeifer and Allen, 2012). More studies are needed to further investigate the role of the reward system in social decision-making. A cluster of activation in the left superior TP was found in the SCDT when choosing the dangerous choice as compared to the safe choice. Primarily conceived as playing a key role in semantic memory, recent reviews show that the TP has also been implicated in social cognition processes including emotion processing, moral cognition, person-specific knowledge, knowledge about social behavior, stereotypes, and ToM (Ferstl and von Cramon, 2007; Olson et al., 2007; Ross and Olson, 2010; Wong and Gallate, 2012). This would suggest that emotional and social processing is also implied in choosing the dangerous option (Blakemore and Robbins, 2012; Crone and Dahl, 2012). An alternate explanation is that the left TP has been more actively engaged in tasks that include a strong verbal component, which is likely to be the case of the SCDT (Mar, 2011). However, this explanation should be ruled out since in our normative studies both verbal choices in risk situations were controlled for semantic and syntactic complexity.

In spite of the small range of ages employed in this study, the adolescents exhibited more activation in two regions in the contrast risk > ambiguous situations. First, adolescents activated the right DLPFC significantly more than young adults. This finding is in line with previous studies using wider ranges of age groups, which also reported more activation in DLPFC areas in children and adolescents than in adults in gambling tasks (Galvan et al., 2006; Van Leijenhorst et al., 2010). According to Steinberg (2008), 2010 considerable evidence suggests that higher level cognition, including the uniquely human capacities for abstract reasoning and deliberative action, is supported by a recently evolved brain system, including highly interconnected regions in the lateral pre-frontal and parietal association cortex and parts of the ACC. Second, adolescents exhibited greater activation than young adults in the right TPJ, a region considered crucial for ToM. Developmental fMRI studies of mental state attribution also showed decreases in ToM regions (e.g., mPFC and right TPJ) between adolescence and adulthood (Blakemore, 2008; Young et al., 2010; Burnett et al., 2011; Sebastian et al., 2011).

When examining developmental effects in the specific contrast dangerous > safe choices in the SCDT, we found that young adults exhibited higher bilateral activations over the MTG and over the right pre-SMA than adolescents. The first result suggests the further engagement of part of the ToM circuitry when choosing the dangerous option as compared to the safe option at later ages. In addition, it was also found that young adults activate the pre-SMA more than adolescents during dangerous choices in the SCDT. The functional role of the pre-SMA has been recently associated with the need to exert complex cognitive control in conflict processing (Nachev et al., 2008; Usami et al., 2013), such as the ones required in free-choice tasks like in SCDT. This cognitive control is suggested to require complex motor skills such as alternation of motor plans, task switching, acquisition of new motor skills, and motor selection. In sum, the process of choosing a dangerous option in young adults seems to involve a further consideration of the social aspects of the risk situation as well as the planning of the action involved in the choice for control purposes.

Concerning the gender differences, only young women exhibited differential activations (female > male) in the risk > ambiguous contrast in SCDT. Specifically, young women elicited more activation in the right insula and the superior temporal gyrus than young men under risky decision-making conditions, suggesting that they probably get more emotionally engaged in the anticipation of aversive outcomes resulting from dangerous options (Clark et al., 2008). Increased insula activity in women is also consistent with the hypothesis that they could be more empathic with the character’s risky decision-making than men, as the role of this region in empathic processing has been well established (Singer et al., 2004, 2006; Botvinick et al., 2005; Jackson et al., 2006; Lamm et al., 2007; Saarela et al., 2007). Right and left TPJ were similarly activated for both genders in our study, however only women activated one region of the ToM network more than men, namely the right superior temporal gyrus, suggesting their further engagement in the modeling of peers’ state of mind. No further gender differences were found when considering the specific contrast dangerous > safe options.

A limitation should be mentioned at this point. Activations in the control and the social cognition networks were not related to individual differences in the risk-taking in the SCDT. This was probably due to the reduced individual variability in the decision-making task, resulting from the proximity of the two age groups and the selection of typical risky scenarios according to the normative studies. In fact, performance in the SCDT was not sensitive to either age- or gender-related differences. This lack of sensitivity limits the ability of the study to explain individual differences in actual risk-taking behavior in social contexts. It also limits sound conclusions regarding the specific role of self-reflection and ToM processes in risk-taking behavior. Future applications of the SCDT to a high-risk group of adolescents and young adults would increase the individual variability, thereby providing further insights into the role of the social cognition processes in social decision-making.

In conclusion, neuroimaging results with the SCDT have demonstrated a differential pattern of activations in neural networks related to cognitive control and social cognition processes in the risk situations compared to the ambiguous situations, even though in both cases a decision is made in a naturalistic scenario including the presence of peers. While the activation of control-related regions was also shown in previous studies of decision-making using a variety of tasks, it appears that the activation of brain regions implied in emotional and social cognition processes is specific to social decision-making tasks in which the presence of peers in the scenario is relevant for the type of decisions being made. Choosing the dangerous option involved a further activation of control areas (ACC) and an emotional and social cognition area (TP), which was not previously reported in decision-making studies. The cognitive control and ToM systems were dependent on the participants’ age in the right DLPFC and right TPJ regions, respectively, towards a more mature functioning. Further engagement in ToM regions (bilateral MTG) and in motor control regions (pre-SMA) related to the planning of actions were found in young adults when choosing the dangerous option. Finally, female participants more than males differentially activated the right insula and right superior temporal gyrus suggesting that they get more emotionally engaged and performed an additional modeling of the peer’s state of mind in the risky decision-making conditions.

Our findings with the SCDT contribute in three ways to the current dual model of adolescent risk-taking based on the existence of a developmental imbalance between the cognitive control and reward brain circuitry (Steinberg, 2008, 2010; Ernst et al., 2009; Somerville et al., 2010; Casey et al., 2011). First, the present activation patterns suggest the engagement of emotional and social cognition processes in risk decision-making, in addition to the cognitive control processes traditionally reported. Second, our developmental findings indicate that control processes in risk decision-making are not the only ones that undergo late developments but also the emotional and social cognition processes (Blakemore and Robbins, 2012; Crone and Dahl, 2012). Finally, our results highlight the need of improving the functional attribution of developmental results (Pfeifer and Allen, 2012). In the same study, we have obtained developmental changes indicating that teens activate the right regions of DLPFC and TPJ more than young adults as well as changes indicating that young adults activate the MTG and the pre-SMA more than adolescents. The notion of neural efficiency, as it reflects the need to engage more brain activity in these areas in the less mature brain, is compatible with the first results but not with the second ones. In sum, the present findings substantiate the call for more comprehensive developmental accounts that incorporate the role of emotional and social cognition processes in risk decision-making in social contexts and refine the interpretation of the developmental changes observed.

## Conflict of Interest Statement

The authors declare that the research was conducted in the absence of any commercial or financial relationships that could be construed as a potential conflict of interest.
